# Risk Factors Addressed in Programs for the Prevention of Child and Adolescent Suicide in the School Setting: A Systematic Review

**DOI:** 10.62641/aep.v53i6.1938

**Published:** 2025-12-17

**Authors:** Adoración Díaz-López, Blanca Tejero-Claver, Juan Faura-García, Hilario Blasco-Fontecilla, Eduardo González-Fraile, Joaquín González-Cabrera

**Affiliations:** ^1^Instituto de Transferencia e Investigación (ITEI), Universidad Internacional de La Rioja (UNIR), 26006 Logroño, Spain; ^2^Faculty of Education and Humanities, Universidad Internacional de La Rioja (UNIR), 26006 Logroño, Spain; ^3^Center of Biomedical Network Research on Mental Health (CIBERSAM), 28029 Madrid, Spain; ^4^Emooti-Instituto de Salud Mental y Bienestar, 28010 Madrid, Spain

**Keywords:** risk factors, prevention, programs, suicide, adolescents

## Abstract

**Background:**

Suicide is increasing among adolescents and young adults worldwide, so its prevention is a topic of great educational interest. In this context, several prevention programs have been developed. However, this problem continues to increase among young people.

**Methods:**

The objective of this study is to systematically analyze the concordance between the risk factors addressed in suicide prevention programs in school settings and the suicide risk factors (RFs) described in the literature (systematic and meta-analyses). Following the Preferred Reporting Items for Systematic reviews and Meta-Analyses (PRISMA) protocol, this review was registered in the International Prospective Register of Systematic Reviews (PROSPERO) with the code CRD42023431649. After launching the search algorithm in the various databases, Web of Science (WOS), SCOPUS, Education Resources Information Center (ERIC), ProQuest Psychology, and PubMed, duplicate references were removed using the bibliographic reference management software Zotero. Two independent researchers assessed their possible eligibility. A third judge resolved any disagreements on the inclusion/exclusion of the selected articles. For the quality assessment, the Joanna Briggs Institute (JBI) was employed. Finally, 24 articles published between January 1, 2000, and February 1, 2025, were selected. The data extraction and qualitative analysis were divided in three phases: (1) Scoping umbrella review of suicide risk factors; (2) Systematic review of suicide risk factors addressed in child and adolescent suicide prevention programs and their efficacy; (3) Systematic analysis of the concordance between suicide risk factors found in the literature of systematic reviews and meta-analyses and their inclusion in prevention programs.

**Results:**

The risk factors more frequently addressed in the programs are anxiety, depression, peer support, and social relationships. Sexual orientation and bullying/cyberbullying are two risk factors whose role in adolescence is crucial and which are barely or not addressed in current prevention programs. Multi-modal interventions provide the best indicators of effectiveness. In addition, the inclusion of working with the family appears to be a component that affects the effectiveness of the programs. A relationship was found between a higher number of risk factors addressed in the programs and their effectiveness.

**Conclusion:**

There is a need to update and create new programs for Generation Z and Alpha students.

## Introduction

Suicide is a complex and multifaceted phenomenon that is usually associated with numerous risk factors (RFs) [1]. Suicide has severe social, economic, psychological, and family repercussions. Although the absolute number of suicides worldwide has shown a slight downward trend, it still causes around 700,000 deaths per year [2]. In addition, self-injurious behaviors (suicidal and nonsuicidal) continue to increase globally. Thus, the number of suicide attempts is 20 times higher than the number of suicides [3].

Despite the worldwide decline in suicide in the general population [4], suicide is increasing among adolescents and young adults worldwide. Suicide is the fourth leading cause of death among young people aged 15 to 29 [4]. In the USA, suicide is the second leading cause of death in children aged 10 to 14 and the third leading cause of death in young people aged 15 to 24 [5]. Moreover, the highest suicide rate among European adolescents is found in Lithuania and Estonia, with rates of more than 11 deaths per 100,000 young people aged 15–19 (2.5 times higher than the European Union’s average). In contrast, the lowest rates are recorded in southern European countries [6].

In this context, and bearing in mind that suicide prevention is possible [7], several prevention programs have been developed targeting the child and adolescent population. It is estimated that a 100% effective intervention could prevent up to 30% of teenage suicides [8, 9]. In this line, as highlighted by Isometsä [10], understanding the clinical and psychosocial risk factors associated with suicidal behaviour is essential for identifying vulnerable individuals and guiding prevention efforts. In this line, following Ati *et al*. [11], one of the first steps to prevention is to determine the RFs that indicate whether an individual, community, or population is particularly vulnerable to suicide. Therefore, the identification of suicide RFs enables the design of tailored and effective evidence-based interventions. Various review studies and meta-analyses have analyzed the most prevalent suicide RFs in both the child and adolescent population [11, 12, 13, 14, 15, 16, 17] and the general population [18, 19, 20, 21, 22, 23, 24]. According to them, the main RFs are: substance use (alcohol, tobacco, others), belonging to minorities that are likely to be rejected (such as a different sexual orientation than the regular one), physical well-being, sleep difficulties and school performance, peer relationships, impulsivity, prior suicidal ideation, previous suicide attempts, eating disorders, anxiety, depression, and antisocial behaviors. These RFs may be addressed in the prevention programs and the promotion of certain values, such as tolerance and respect for others and their differences.

Suicide prevention actions can be developed in different contexts, but following the recommendations of the World Health Organization [4], the school setting is an environment particularly conducive to acquiring socio-emotional competencies. For this reason, this setting appears to be the optimal ecological niche for implementing preventive actions [25]. In this sense, numerous programs implemented in the school context, mainly in the USA and Europe, have proven effective. These programs can be classified into programs of awareness-raising, information, training, development, screening, therapeutic interventions, and multi-modal interventions [26]. Along these lines, it is worth noting that the first prevention programs in the school setting emerged in the 2000s and were primarily of the training type, such as the Coping and Support Training, Care, Assess, Respond, Empower (C-CARE CAST) [27] or the Psychoeducational program [12]. In 2010, multi-modal programs (including two or more programs or strategies) began to emerge, such as the Saving and Empowering Young Lives in Europe (SEYLE) [9] or the Signs of Suicide (SOS) program [28]. In addition, there are other programs, such as the Multi-modal stepped-prevention program [29], Standard Protocol Items: Recommendations for Intervention Trials (SPIRIT) [30], or Du und deine Emotionen (DUDE) [31], whose implementation has not yet been carried out, and only the protocol has been designed.

Despite the number of existing prevention programs, child and adolescent suicide rates are very high, and have increased in recent years in many geographical areas [32]. To date, despite the existence of review studies on the effectiveness of child and adolescent suicide prevention programs [17, 33, 34, 35, 36], only two review studies have analyzed their contents [17, 34]. Still, none of them delve into the RFs addressed in the programs. Therefore, a gap exists in the literature that may have practical implications for the development of new programs or the revision of existing ones. The main objective of this systematic review is to analyze the degree of fit between suicide RFs reported in the literature (systematic reviews and meta-analyses) and the RFs addressed in school-based suicide prevention programs for children and adolescents. Likewise, we will study those RFs included in the intervention programs that show greater efficacy in the prevention of child and adolescent suicide. The specific goals are:

1. To carry out a scoping umbrella review of the published systematic reviews and meta-analyses of suicide RFs in the general and child and adolescent populations, extracting those factors that can potentially be addressed (by an intervention or program).

2. To conduct a systematic review of published empirical studies on the efficacy of suicide prevention programs in children and adolescents in schools.

3. To systematically analyze the technical characteristics and contents addressed in suicide prevention programs found in the previous step.

4. To systematically extract which RFs are addressed in the suicide prevention programs found, and their degree of agreement with those found in the umbrella review.

5. To analyze the effectiveness of the programs in reducing those RF.

## Method

### Protocol 

We conducted a systematic review following the PRISMA guidelines [37, 38] to achieve the above objectives. This review was registered in the International Prospective Register of Systematic Reviews (PROSPERO) with code CRD42023431649.

### Sources and Data Search Strategy

#### Search Method

The search was conducted in five electronic databases: WOS, SCOPUS, ERIC, ProQuest Psychology, and PubMed. The search was conducted using the algorithm described below and was limited to articles published since 2000.

#### Search Terms

Educational Programs: (Educational Programs OR Extracurricular Programs OR Educational Program Evaluation OR Educational Program OR School Intervention).

School Context: (School* OR Classroom* OR Classes OR Classical OR University* OR Course*).

Intervention/Prevention: (Program* OR Intervention* OR Preventive OR Prevention OR Prevent).

Self-Destructive Behaviors: (Self-Destructive Behavior OR Self-Injurious Behavior OR Suicide OR Suicidality OR Suicidology OR Suicidal Behavior OR Self-Injury).

Target Population: (Youth OR Young Person OR Adolescent OR Child OR School-Aged Adolescent OR Adolescents).

#### Boolean Operators

Boolean operators were used to combine different search terms effectively:

AND: Used to combine different concepts and ensure that the results included all relevant elements (e.g., “educational programs AND school* AND self-injury”).

OR: Used within each group of related terms to cover synonyms or variations (e.g., “self-destructive behavior OR self-harm”).

As of February 1, 2025, five electronic databases (WOS, SCOPUS, ERIC, ProQuest Psychology, and PubMed) were examined using the following final search algorithm: (Educational programs OR After school programs OR educational program evaluation OR educational program OR school based intervention) AND (school* OR classroom* OR classes OR classical OR college* OR course*) AND (program* OR intervention* OR preventive OR prevention OR prevent) AND (Self-destructive behavior OR Self-injurious behavior OR suicide OR suicidal OR suicidality OR suicidology OR suicide behavior OR self-harm) AND (young OR youth OR adolescent OR child OR scholar teen OR teenagers). A time limit was applied from the year 2000 onwards.

### Eligibility Criteria

Eligibility criteria were established following the PICOS framework (Population, Intervention/exposure, Comparator, Outcome, and Study) as follows: (1) Population: Any participant of either sex under 18 years of age. As an exception, studies whose participants were 19 years old were accepted, as long as they were students who were pursuing non-university studies in schools; studies whose scope of application was a formal non-university school setting; (2) Intervention or exposure: prevention programs in schools settings; (3) Comparator: no specified comparator; (4) Outcome: main outcomes were suicidal ideation and suicidal behaviors. Secondary outcomes were the rest of the RFs addressed in the scoping umbrella review; (5) Study: any interventional study design (experimental or quasi-experimental) using quantitative data. The exclusion criterion was studies with samples of individuals with some psychiatric pathology or mental health diagnosis. This criterion was established because interventions focused on severe mental illness in children often include pathology-specific content. This may lead to heterogeneity of results and pose difficulties in synthesizing the results.

### Study Screening and Selection Process

After launching the search algorithm across different databases, duplicate references were removed using the bibliographic reference management software Zotero (version 7.0; Corporation for Digital Scholarship, Fairfax, VA, USA) [39]. Two independent and blinded researchers assessed their possible eligibility based on the title and abstract. A third independent and blinded judge resolved any disagreements regarding the inclusion/exclusion of the selected articles. The references selected, based on the title and abstract, were again evaluated in full text. After identifying the studies to be included in the review, we extracted the relevant information from each one using a template designed for this purpose.

### Quality Assessment

We used the Joanna Briggs Institute (JBI) Critical Appraisal Checklists to assess the risk of bias (methodological quality of studies) [40]. This is an eight-item checklist with four response options (“Yes”, “No”, “Unclear”, or “Not applicable”). Only “Yes” is scored with 1 point (while the others score 0 points). The total score ranges from 0 to 8. Although there are no explicit cut-off points, a common practice is to consider studies as high quality if they meet more than 80% of the criteria, moderate quality if they meet 50 to 80%, and low quality if they meet less than 50% [41].

## Data Extraction and Qualitative Analysis

For an orderly and structured process, this research was carried out in three sequenced phases to fulfill the research objectives:

### First Phase: Scoping Umbrella Review Analysis of Suicide RFs 

To carry out an orderly, coherent, and thorough analysis of the state of the art, an analysis of the systematic reviews and meta-analyses of suicide RFs of the last two decades (2000–2025) was performed through a scoping umbrella review. All potentially addressable suicidal ideation RFs found were extracted and, according to their nature, organized into four types of RF: individual, psychological, family, and social. This categorization aligns with the approaches of major health organizations [4, 42] and academic literature [9, 43], providing an effective and understandable framework for analyzing and preventing suicide. These factors were then grouped into two categories, according to their degree of occurrence in the different review studies and meta-analyses: (a) high-relevance factors (those that appeared in 3 or more reviews or meta-analyses) and (b) medium-relevance factors (those that appeared in only 1 or 2 reviews or meta-analyses).

### Second Phase: Systematic Review of Suicide RFs Addressed in Child and Adolescent Suicide Prevention Programs 

The fundamental characteristics of the studies and the features of the suicide prevention programs identified in the selected articles, along with their technical aspects, were analyzed. In this sense, during the process, we contacted the creators of the analyzed programs by email and/or through the official websites of the programs to request information about the programs’ contents. There were two ways to report the requested information: (1) authors were asked to offer us access to the programs (through a 24-hour license or for a limited time) so that we could justifiably check which factors are addressed; (2) a questionnaire was sent with the possible RFs so that the authors could indicate those addressed in their program. The authors were contacted for the first time at the beginning of September 2023, and a reminder was sent to them at the end of the month. Forty per cent of the authors agreed to participate. The authors of this review had access to two programs and received information from three additional programs.

### Third Phase: Systematic Analysis of the Concordance Between Suicide RFs Found in the Literature of Systematic Reviews and Meta-Analyses and Their Inclusion in Prevention Programs

We compared and analyzed the degree of agreement between the information on suicide RFs addressed in each program and the RFs more prevalent in the literature (obtained in phase one).

## Results

### Scoping Umbrella Review Analysis on Suicide Risk Factors

We analyzed a total of 15 systematic review studies or meta-analyses examining suicide RFs. A total of 26 RFs were found: 13 of high relevance and 12 of medium relevance (Table 1, Ref. [11, 12, 13, 14, 15, 16, 17, 18, 19, 22, 23, 24, 44, 45, 46]).

**Table 1.** **Suicide risk factors found in the scoping umbrella review**.

Categories	Relevance	
	High	Medium
Individual factors	Sexual orientation [12, 13, 14, 16]	Tobacco use [13, 15]
	Substance abuse [11, 12, 13, 15, 19]	Overall physical health and well-being [11, 13]
	Alcohol consumption [13, 15, 19]	Difficulty sleeping [11, 23]
		School performance [13, 15]
		Mobile use [11]
Psychological factors	Anxiety [12, 13, 15, 18, 22]	Self-esteem [13, 17]
	Depression [13, 15, 18, 19, 24]	Problem-solving techniques [11, 19]
	Previous suicide attempt [12, 14, 18]	Impulsivity problems [19]
	Hopelessness [18, 19, 24]	Prior thoughts of suicide [15]
		Eating disorders [13]
		Emotional intelligence [19]
Family factors	Communication & family relationships [11, 12, 13, 15, 19]	
	Suicidal behaviors in family members [12, 13, 14, 15]	
Social factors	Exposure to suicide events in the media [12, 13, 14, 19]	Suicide of a friend [13]
	Antisocial behaviors/peer bullying [11, 12, 13, 18, 19]	
	Peer relationships [13, 14, 19]	
	Cyberbullying [44, 45, 46]	

In this sense, the factors that load higher in the analyzed studies and which, therefore, should have a greater weight in preventive actions are: Sexual orientation and substance abuse; Psychological factors: anxiety and depression; Family-factors: suicidal behavior in family members and family communication and relationships; and Social factors: exposure to suicide events in the media and antisocial behaviors/peer bullying (see Table 1).

### Study Selection

**Fig. 1.**
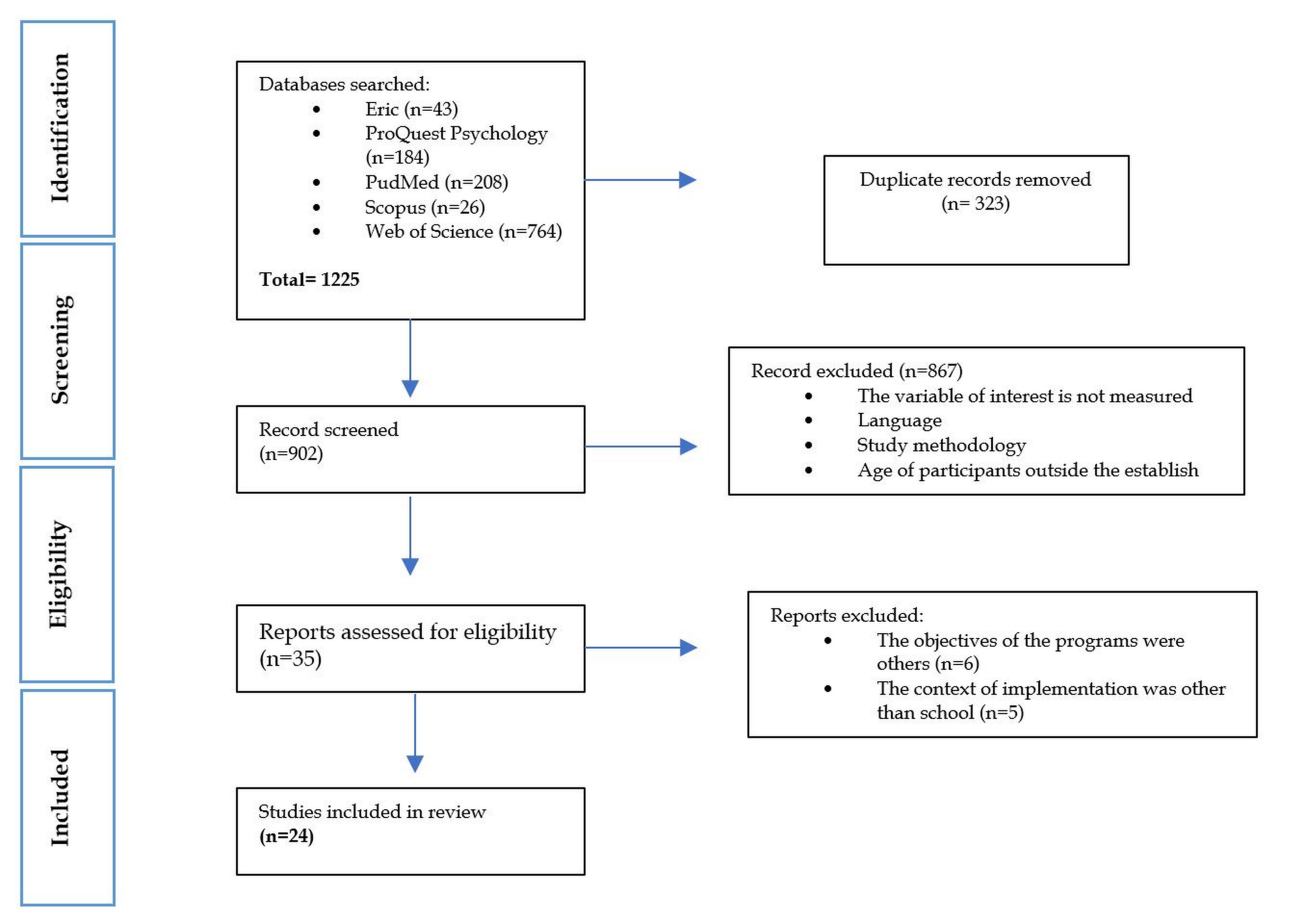
**Flowchart-PRISM declaration of the study selection process**.

One thousand one hundred twenty-five publications were identified. Two hundred twenty-three duplicate references were removed. A total of 902 references were reviewed by title and abstract. Eight hundred sixty-seven references were excluded for various reasons (lack of evaluation of variables of interest, study methodology, population outside the established age range). Thirty-five references were evaluated in full text. The initial degree of agreement among the reviewers was 92%. After discussing their eligibility with the third reviewer, a 100% agreement was reached. Finally, 24 articles were selected. A summary of the study selection process is presented in the PRISMA flowchart (see Fig. 1).

### Study Characteristics

Our search identified 24 articles. All the studies used a quasi-experimental design. Ten studies were conducted in the United States of America [27, 28, 47, 48, 49, 50, 51, 52, 53, 54], three in Australia [55, 56, 57], two in Chile [58, 59], in Europe [9, 60, 61, 62, 63, 64, 65] and two in Canada [66, 67]. The participants’ ages ranged from 11 to 19 years old. The total sample size, including all the studies, was 52,222. The setting was public schools in all cases. All the studies included a follow-up at 3 or 6 months. All the studies included presented a high-quality assessment. Study characteristics are summarized in Table 2 (Ref. [9, 27, 28, 47, 48, 49, 50, 51, 52, 53, 54, 55, 56, 57, 58, 59, 60, 61, 62, 63, 64, 65, 66, 67]).

**Table 2.** **Characteristics of included studies**.

Author, year	Country	Program	Sample size	Mean Age	Sex (%)	Setting	Follow-up	Quality assessment
Wasserman *et al*. (2015) [9]	Europe	SEYLE	11,110	14–15 years	M-40.8 F-59.2	Public schools	Yes	H
Thompson *et al*. (2001) [27]	USA	C-CARE CAST	460	14–19 years	M-48.0 F-52.0	Public schools	Yes	H
Aseltin and DeMartino (2004) [28]	USA	SOS	2100	14–18 years	M-49.5 F-50.5	Public schools	Yes	H
Aseltine *et al*. (2007) [47]	USA	SOS	4133	14–18 years	M-50.9 F-49.1	Public schools	Yes	H
Clark *et al*. (2022) [48]	USA	SOS	2537	11–14 years	M-51.0 F-49.0	Public schools	Yes	H
Schilling *et al*. (2016) [49]	USA	SOS	3120	11–18 years	M-51.0 F-49.0	Public schools	Yes	H
Lindow *et al*. (2020) [50]	USA	YAM	1878	12–17 years	M-51.9 F-48.1	Public schools	Yes	H
Freedenthal (2010) [51]	USA	Yellow Ribbon	146	11–18 years	M-40.0 F-60.0	Public schools	Yes	H
Flynn *et al*. (2016) [52]	USA	Yellow Ribbon	3257	11–18 years	M-49.0 F-51.0	Public schools	Yes	H
Walker *et al*. (2009) [53]	USA	LifeSavers	63	14–17 years	NA	Public schools	No	H
Wyman *et al*. (2010) [54]	USA	Sources of Strength	2675	15–16 years	M-48.0 F-52.0	Public schools	Yes	H
McGillivray *et al*. (2021) [55]	Australia	YAM	556	13–16 years	M-43.4 F-56.6	Public schools	Yes	H
Hart *et al*. (2020) [56]	Australia	Teen Mental Health	1605	15–17 years	M-55.3 F-44.7	NA	Yes	H
Hart *et al*. (2019) [57]	Australia	Teen Mental Health	475	12–15 years	NA	Public schools	Yes	H
Gaete *et al*. (2025) [58]	Chile	Reframe-IT+	52	14–20 years	M-23.1 and F-76.9	Public schools	No	H
Nuñez *et al*. (2024) [59]	Chile	Reframe-IT+	1546	9–11 years	NA	Public schools	Yes	H
Baggio *et al*. (2022) [60]	Switzerland	Brief universal	305	14–29 years	M-44.2 F-55.8	Public schools	Yes	H
Portzky and van Heeringen (2006) [61]	Belgium	Psychoeducational program	172	14–18 years	M-38.3 F-62.7	Public schools	Yes	H
Baetens *et al*. (2024) [62]	Belgium	School-Based Early Intervention	329	11–14 years	M- 44.4 F-55.6	Public schools	Yes	H
Díez-Gómez *et al*. (2024) [63]	Spain	PositivaMente	264	14–15 years	M-45.4 F-54.5	Public schools	Yes	H
Barzilay *et al*. (2019) [64]	EU	YAM	11,110	14–15 years	M-41.0 F-59.0	Public schools	Yes	H
Kahn *et al*. (2020) [65]	EU	YAM	3602	14–16 years	M-42.8 F-57.2	Public schools	Yes	H
Silverstone *et al*. (2015) [66]	Canada	EMPATHY	3244	11–18 years	M-51.7 F-48.3	Public schools	Yes	H
Silverstone *et al*. (2017) [67]	Canada	EMPATHY	3244	11–18 years	M-51.7 F-48.3	Public schools	Yes	H

Note: SOS, Signs of Suicide; YAM, Youth Aware of Mental Health; SHEILE, Suicide Prevention in European Youth; H, high quality; NA, Not Available.

### Characteristics of Suicide Prevention Programs

Before analyzing the contents of child and adolescent suicide prevention programs in the school setting, it is pertinent to explore some general data from the 13 programs applied in the 24 selected publications to provide a global and contextualized picture of the state of the art (Table 3, Ref. [9, 26, 27, 53, 54, 57, 59, 60, 61, 62, 63, 66, 68, 69, 70]).

**Table 3.** **Technical characteristics of child and adolescent suicide prevention programs**.

	Program name	Program authors	Country/Continent of origin	Program dynamics	Number of sessions and session duration	Age of recipient	Teaching Professional	Requires training	Working with family	Program access	Type of program according
1	YAM	Wasserman *et al*. (2015) [9]	Sweden	It consists of bringing together several young people from the same school to talk about issues related to mental health. Role-plays and debates are held to discuss everyday situations about stressors and mental health issues.	7 sessions Session duration: 50 min	13–17 years	Two specifically trained adults (YAM instructors) with experience working with youth groups	Yes	No	Yes	Training in psychoeducation
2	Psycho-educational program	Villalobos-Galvis *et al*., (2023) [26] & Portzky and van Heeringen (2006) [61]	Belgium	Psychoeducational programs focusing on knowledge, attitudes, suicide coping styles, and levels of hopelessness.	1 session Session duration: 120 minutes	14–18 years	Psychologist of the Unit for Suicide Research	Yes	No	No	Training in psychoeducation
3	C-CARE CAST	Thompson *et al*. (2001) [27]	USA	The program begins with an individual assessment interview followed by a counseling and social “connections” intervention session with parents and school staff. It is completed with a skills-training program.	12 sessions Session duration: 120-minute interview and 60-minute training sessions	14–19 years	Independent supporting professional interviewers, principal investigator, program coordinator	Yes	Yes	No	*Gatekeeper *or watchman training
4	LifeSavers peer-support suicide prevention	Walker *et al*. (2009) [53]	USA	The training is provided through “listening circles”, in which students learn to express thoughts and feelings, maintain confidentiality, and demonstrate sensitivity to others.	3 days	Secundaria	Teachers, counselors, coaches or LifeSaver counselors	Yes	No	No	*Gatekeeper *or watchman training
5	Sources of Strength	Wyman *et al*. (2010) [54]	USA	The program consists of 3 phases: The first includes the training of staff as advisors. The second consists of interactive training in protective factors, problem-solving skills, and help-seeking. In the latter, leaders encourage identifying trusted adults and spreading messages about sources of strength.	3 sessions Session duration: 20–30 minutes	12–16 years	Certified trainers	Yes	No	Yes	Multi-modal interventions
6	Teen Mental Health First Aid	Hart *et al*. (2019) [57]	Australia	This program seeks to teach mental health literacy, reduce stigma, and encourage help-seeking and prevention. A self-recorded questionnaire is applied to assess suicide RFs, assessed by mental health professionals.	3 sessions Session duration: 75 minutes	15–17 years	External instructors	Yes	No	Yes	Training in psychoeducation
7	Reframe-IT+,	Nuñez *et al*. (2024) [59]	Chile	This study aimed to test the effectiveness of a blended intervention (Reframe-IT+), based on the Cognitive-Behavioral Model, to reduce suicidal ideation.	13 sessions	9–11 years	Trained psychologists	Si	No	-	Multi-modal interventions
8	BUSP	Baggio *et al*. (2022) [60]	Switzerland	The program includes a lecture, a discussion group based on case studies, a quiz on myths and facts about suicide, and an illustrated book. It provides general information about suicidal behavior, helps identify RFs, and warns about signs of suicidal intent.	1 session Session duration: 90 minutes	14–19 years	Specialist in suicide prevention or psychologist	Yes	No	Yes	Multi-modal interventions
9	School-Based Early Intervention	Baetens *et al*. (2024) [62]	Belgium	This study aims to evaluate the efficacy of a universal prevention program in schools for NSSI and mental complaints while enhancing resilience and mental health in 11–14-year-old adolescents. It is a universal 4-hour classroom prevention, with a focus on emotion regulation, mental health, and specific strategies to prevent NSSI and reduce stigma.	4 h classroom 15 minutes per student (individualy)	11–14 years	3 conselors	No	No	No	Multi-modal Intervention
10	PositivaMente	Díez-Gómez *et al*. (2024) [63]	Spain	The program uses videos, rol playing etc., in order to promote wellness. It has 5 modules: Module I: awareness, Module II: risk factors and protective factors, Module III: stress and crisis, and Module IV: thinking and emotion.	11 sessions Session duration: 45 minutes	14–15 years	Teachers	No	No	No	Multi-modal interventions
11	EMPATHY	Silverstone *et al*. (2015) [66]	Canada	The group of high-risk students is identified with whom an online cognitive-behavioral therapy program is conducted, as well as several interactions with the institution’s trained staff. Cognitive-behavioral therapy (CBT) is applied to improve resilience and depression.	8 sessions Session duration: 45 minutes	11–18 years	Resiliency Coaches	Yes	Yes	No	Multi-modal interventions
12	SOS	Mindwise Innovations (1999) [68]	USA	The program features videos dramatizing others’ suffering, and discussions of the videos are conducted so that students learn to detect the signs of suicide in themselves and others as an emergency.	3 sessions Session duration: Between 45–60 minutes	11–18 years	School counselors and social work staff	No	Yes	Yes	Multi-modal interventions
13	SEYLE	Wasserman *et al*. (2012) [69]	Europe	This program comprises three parts: (1) Question, Persuade, and Refer (QPR) to train teachers and other staff. (2) Youth Aware of Mental Health (YAM) Handout, six educational posters. (3) Screening by Professionals (ProfScreen) program: intervention through interactive workshops and talks on mental health.	5 sessions Session duration: 60 minutes	14–15 years	Trained Instructors	Yes	No	No	Multi-modal interventions
14	Yellow Ribbon	Hidman (2008) [70]	USA	It involves training during classes, after which the student is given a wristband that includes the three steps they should take to help themselves or others at risk of suicide, as well as a list of phone numbers for a national helpline.	6 sessions Session duration: 60 minutes.	11–18 years	2 suicide prevention experts, two school counselors, and the principal	Yes	No	No	Information strategies

Firstly, concerning the origin of the programs, they are primarily preventive actions in the USA (SOS, YELLOW RIBBON, C-CARE CAST, LifeSavers peer-support suicide prevention, and Sources of Strength). Likewise, the time frame for the publication of the programs ranges from 2001 for the C-CARE-CAST program to 2025, for Internet-based Cognitive Behavioural Therapy Program for Suicidal Adolescents (REFRAME-IT).

As for the structure of the programs, the shortest one consists of a single session (Psychoeducational program), while the longest one comprises 12 sessions (C-CARE CAST), with an average duration of almost six sessions for the programs. Concerning session duration, the shortest is 20 minutes (Sources of Strength) and the longest is 2 hours (Psychoeducational Program), averaging 70.35 minutes. Additionally, only the SOS program can be taught without training, while the rest of the programs require a qualified professional to implement them. Another highlight is that only two programs work with the family (SOS and C-CARECAST). Finally, multi-modal interventions are the most common type of programs (SOS, EMPATHY, Youth Awareness Mental Health (YAM), Brief Universal Suicide Prevention (BUSP), Sources of Strength, School-Based Early Intervention, PositivaMente, REFRAME-IT+).

### Analyze Suicide RFs Addressed in Prevention Programs

The content of the programs was analyzed, and the RFs of high and medium relevance addressed in them were extracted (Table 4, Ref. [9, 27, 28, 47, 48, 49, 50, 51, 52, 53, 54, 55, 56, 57, 58, 59, 60, 61, 62, 63, 64, 65, 66, 67], Table 5, Ref. [9, 27, 50, 51, 52, 53, 54, 55, 56, 57, 58, 59, 60, 61, 62, 63, 64, 65, 66, 67]).

**Table 4.** **Highly relevant factors addressed in suicide prevention programs**.

Name of the program	Individual			Psychological				Family		Social				Total
	Sexual orientation	Substances abuse	Alcohol consumption	Anxiety	Depression	Previous suicide attempt	Hopelessness	Communication & family relationships	Suicidal behaviors in family	Exposure to suicide in media	Antisocial behaviors/peer bullying	Peer relationships	Cyberbullying	
SOS [28, 47, 48, 49]	✓	✓	✓	✓	✓	✓	✓	✓	✓					9
YAM [50, 55, 64, 65]				✓	✓	✓	✓	✓		✓		✓		7
EMPATHY [66, 67]		✓	✓	✓	✓	✓								5
SEYLE [9]		✓	✓	✓	✓	✓	✓	✓				✓		8
BUSP [60]		✓	✓	✓	✓	✓	✓		✓		✓	✓		9
Yellow Ribbon [51, 52]						✓								1
Teen Mental Health First Aid [56, 57]				✓	✓	✓	✓	✓						5
Psychoeducational Program [61]					✓	✓	✓					✓		4
C-Care Cast [27]		✓		✓	✓	✓	✓							5
Lifesavers [53]														0
Sources Of Strength [54]												✓		1
School-Based Early Intervention [62]				✓	✓	✓		✓				✓		5
PositivaMente [63]					✓	✓		✓			✓	✓		5
Reframe-IT+ [58, 59]				✓	✓	✓	✓	✓	✓		✓	✓		8

Note: BUSP, Brief Universal Suicide Prevention program for school-aged youths; Dark gray Cell, Significant improvement; Light gray Cell, no significant changes; ✓included.

**Table 5.** **Medium relevance RFs addressed in suicide prevention programs**.

Name of the program	Individual					Psychological						Social	Total
	Tobacco use	Physical health and well-being	Difficulty sleeping	School performance	Mobile use	Self-esteem	Problem-solving techniques	Impulsivity problems	Prior thoughts of suicide	Eating disorders	Emotional intelligence	Suicide of a friend	
SOS [64, 65]		✓	✓	✓	✓				✓			✓	6
YAM [50, 55, 64, 65]		✓				✓			✓			✓	4
EMPATHY [66, 67]	✓						✓		✓				3
SEYLE [9]		✓				✓			✓			✓	4
BUSP [60]			✓	✓		✓			✓	✓	✓	✓	7
Yellow Ribbon [51, 52]									✓			✓	2
Teen Mental Health First Aid [56, 57]									✓			✓	2
Psychoeducational Program [61]									✓			✓	2
C-Care Cast [27]							✓	✓	✓				3
Lifesavers [53]						✓						✓	2
Sources Of Strength [54]				✓					✓			✓	3
School-Based Early Intervention [62]				✓					✓		✓	✓	4
PositivaMente [63]				✓		✓			✓		✓	✓	5
Reframe-IT+ [58, 59]				✓		✓			✓		✓	✓	5

Note: BUSP, Brief Universal Suicide Prevention program for school-aged youths; ✓included; dark gray Cell, Significant improvement; Light gray Cell, no significant changes.

Concerning the individual factors, the most frequently addressed in the programs are substance use in five programs (SOS, EMPATHY, SEYLE, BUSP, and C-CARE CAST) and alcohol use in four programs (EMPATHY, SEYLE, SOS and BUSP), while least addressed factor is sexual orientation (SOS).

With regard to psychological factors, the most frequently addressed in the programs are anxiety (including stress as a manifestation) and depression. They are present in ten programs (SOS, EMPATHY, SEYLE, BUSP, Teen Mental Health First Aid, C-CARE CAST, School-Based Early Intervention, REFRAME IT+ and YAM) according to the study by Högberg *et al*. [71]. Only depression in the Psychoeducational program. The least addressed psychological factor is hopelessness in eight programs.

The social and family factors most frequently addressed are the relationship with peers in eight programs (YAM, POSITIVAMENTE, REFRAME IT+, SEYLE, Psychoeducational program, BUSP, Sources of Strength, School-Based Early Intervention) and the relationship with the family in seven programs (YAM, SEYLE, Teen Mental Health First Aid, SOS, School-Based Early Intervention, PositivaMente and REFRAME IT+). The least addressed are bullying, which was only addressed in three programs (BUSP, PositivaMente and REFRAME IT+), and cyberbullying, which was not addressed in any program.

On the other hand, concerning the factors of medium relevance (Table 5), in terms of individual factors, the most frequently addressed in the programs are school performance, in six programs (SOS, BUSP, Sources of Strength, School-Based Early Intervention, PostivaMente and REFRAME IT+) and physical health in three programs (SOS, YAM, and SEYLE). On the other hand, the least addressed individual factors are the use of mobile phones and social networks (only in SOS) and tobacco consumption (only in EMPATHY). The most frequently addressed psychological factors are prios thoughts of suicide in all the programs apart form Lifesvers. The only social factor of medium relevance is the suicide of friends (addressed in 12 programs).

## Degree of Agreement Between Suicide RFs Found in Review Analyses and Suicide RFs Addressed in Prevention Programs

Firstly, with regard to individual factors, the ones that load higher in the review studies analyzed and which, therefore, should have a greater weight in preventive actions are sexual orientation [1] and substance abuse [11, 12, 13, 15, 19]. In this sense, the results of the present systematic review partially coincide with the review literature because they report that the individual factor most addressed in the programs is substance use. However, sexual orientation is the least prevalent in the interventions. Regarding psychological factors, it was found that those addressed in prevention programs align with the findings in the scoping umbrella review, with anxiety [12, 13, 15, 18, 22, 58, 59, 62] and depression [13, 15, 18, 19, 24, 58, 59, 63] being the most prevalent both in the literature and in the interventions. Concerning family-type factors, suicidal behaviors in family members [12, 13, 14, 15] and family communication and relationships [11, 12, 13, 15, 19] are the most prevalent in the review studies. These results partially coincide with those found in the present systematic review, in which the family factor most frequently addressed in the school programs is the relationship with the family. At the social level, according to the scoping umbrella review, the factors that have a greater weight in suicidal behavior are exposure to suicidal events in the media [12, 13, 14, 19] and antisocial behaviors and peer bullying [11, 12, 13, 18, 19, 58, 59, 63]. In this sense, the results of the analysis of the content of the programs point to the relationship with peers as the most frequently addressed factor, but also show that bullying and cyberbullying are the least addressed.

## Analysis of the Effectiveness of the Programs in Reducing Suicidal Ideation and Suicide Attempts

For a better understanding of this section, it should be noted that comprehensive prevention refers to those programs that, after implementation, have reported lower prevalence of suicidal ideation and suicide attempts. Meanwhile, partial prevention refers only to a lower prevalence of suicidal ideation after the intervention.

When analyzing the effectiveness of the different programs (see Tables 4,5), on the one hand, we observed that eight of the 14 programs reported overall prevention (lower prevalences of suicidal ideation and suicide attempts) after their implementation (C-CARE-CAST, SOS, YAM, EMPATHY, SEYLE, REFRAME IT+, PositivaMente and School-Based Early Intervention). The RFs common to these programs are: anxiety and depression. In addition, three programs reported partial prevention (lower prevalence of suicidal ideation) after the intervention (Psychoeducational program, BUSP, and Sources of Strength). Likewise, eight programs that reported total or partial evidence were multi-modal. In contrast, two of the programs did not report either overall or partial efficacy (Yellow Ribbon, and LifeSavers peer-support suicide prevention). These programs involved information-strategies, psychoeducation, and training (respectively). Another aspect to consider is the role of family involvement in the effectiveness of programs. Only two programs (SOS and C-CARECAST) worked with the family, among those that reported complete efficacy.

## Numerical Summary of Common RFs Evidence Found in the Different Implementations of Each Program

Programs addressing a higher number of RFs (12 or more) showed a significant reduction in both suicidal ideation and suicide attempts in their samples. These programs were: SOS, SEYLE, School-Based Early Intervention, PositivaMente, Reframe-IT+ and BUSP (the BUSP significantly reduced only suicidal ideation). It should also be noted that these three programs are multi-modal. The programs that address the fewest risks are: LifeSavers peer-support suicide prevention and Yellow Ribbon. These do not present evidence for total or partial suicide prevention.

## Discussion

In this systematic review, we mainly analyzed the degree of agreement between the most relevant RFs in the literature on suicide and the RFs addressed in suicide prevention programs in schools. Our study highlights numerous relevant findings. In the first place, partial concordance was found between the suicidal RFs prevalent in the scientific literature and the suicidal RFs addressed in the prevention programs. Thus, the following RFs are common in the most effective interventions: anxiety, depression and social relationships. Second, we found a relationship between a higher number of suicidal RFs addressed in prevention programs and their effectiveness. Third, multi-modal interventions appear to provide the best indicators of effectiveness. Fourth, the inclusion of working with families appears to be related to the program’s effectiveness. Finally, a salient finding is that there are two RFs (respect for sexual orientation and bullying) that, despite appearing to be critical in preventing child and adolescent suicide nowadays, are barely or not addressed in current prevention programs. Also, there are no programs aimed at preventing Internet risks.

Delving into this last finding, as noted, there is a partial agreement between the suicide RFs found in the scoping umbrella review and those addressed in suicide prevention programs. Thus, to a large extent, the psychological and family factors addressed in suicide prevention programs match the RFs reported in the different review studies. However, relevant discrepancies have been found in individual and social factors. In this sense, it is striking that acceptance and respect for sexual orientation are only addressed in one prevention program despite being a widely verified RF in the literature on suicide. In addition, for adolescents, sexuality is one of the main aspects for the development of their personality and self-esteem [72], and the prevalence of suicide in the LGTBI community is significantly higher, as in the minorities most affected by mental health problems related to stigma and discrimination [73]. One possible interpretation is that most suicide prevention programs are American, and American society has numerous religions in which non-normative sexual orientation may be taboo [74]. Likewise, in terms of social factors, bullying is only addressed in one program, and none of them address cyberbullying. One potential cause could be the limited integration of comprehensive mental health and social risk factors into existing intervention programs. Often, programs tend to focus on a narrow set of factors due to logistical challenges or the prioritization of immediate, measurable outcomes [34]. Additionally, some programs may not have the resources to address emerging issues, such as cyberbullying, which requires specialized strategies and training for educators and students [75]. These results are alarming when considering that several review studies have recorded the relationship between bullying and cyberbullying maintained over time and hopelessness, loss of quality of life, and suicide attempts in adolescents [44, 45, 46]. This lack of content about the manifestations of school violence and sexual diversity in prevention programs is particularly concerning when considering the interrelationship between the two factors. In this sense, adolescents consider homosexuality to be the main reason for bullying or cyberbullying, and the figures for bullying and cyberbullying in the homosexual population are twice those of the heterosexual population [76].

On the other hand, young people in today’s society point to cyberspace as the primary option for leisure and interaction with others [76]. Along these lines, there is a discrepancy between the main problems and risks that may arise in the online setting for adolescents and the contents addressed in suicide prevention programs. Thus, individual factors such as the use of mobile devices do not seem to have a great prominence either in the literature on suicide RFs or among the contents of prevention programs. They are addressed in only one study. Along these lines, it is striking that no program explicitly addresses internet risks, even though all of them are related to severe mental health problems and loss of quality of life in adolescents, which, in turn, are directly related to suicidal ideation and suicide attempts [77, 78]. Specifically, review studies have linked general problematic internet use to low quality of life [79, 80], sexting with anxiety, depression [81, 82], suicidal ideation [83], and cyberbullying with severe mental health problems [84], including self-harm and suicidal behaviors [44, 45, 85, 86, 87]. In addition, other studies report a loss of quality of life in minor victims of cyberdating [88] and in individuals who present several overlapping Internet risks [89]. Likewise, the problematic use of social media is related to severe psychological distress, bodily self-esteem issues [90], anxiety, depression [91], cyberbullying, and suicide attempts [92].

Regarding the effectiveness of prevention programs, the results suggest that, according to the types proposed by [26], the type of intervention may be a determining factor in a program’s effectiveness. Thus, the results show that three of the five programs that reported overall efficacy and two of the three programs that reported partial efficacy were multi-modal interventions. A possible explanation for this fact is that, according to [71], psychological therapy with a multi-modal treatment approach is useful for treating suicidal children and youth. Consequently, following [93], group prevention programs that adopt this approach are effective in increasing short-term attitudes toward suicide and reducing rates of suicide. Another noteworthy aspect regarding the effectiveness of the programs is the role of family participation. Thus, two programs that reported overall effectiveness were the only programs that worked with the family. This may be due, at least in part, to the fact that interventions involving families to promote help-seeking have a positive effect on children [94, 95].

In a more specific analysis, we found that the common risks addressed in programs with proven global efficacy are anxiety and depression. Both psychological factors are the most strongly related to child and adolescent suicide [22, 24] and, therefore, working on them (together with the normalization of help-seeking) can have a significant impact on the program’s effectiveness. In terms of programs with proven partial effectiveness, support or social relationships are identified as the common factor. In adolescence and early youth, the sense of belonging to a group improves self-esteem and mental health [96]. In addition, one epidemiological study concludes that greater perceived social support decreases the likelihood of suicidal ideation in adolescents [97].

In relation to the above, when considering the numerical summary of the factors addressed in each program, it is of particular interest that those programs that address a greater number of RFs are the ones that present greater effectiveness in the overall and partial prevention of suicide. Conversely, programs addressing fewer RFs do not provide evidence of total or partial prevention. In this sense, this finding is consistent with the reports by [4], which indicate that understanding and addressing suicide RFs can help prevent suicide attempts through the development of more efficient programs.However, the cataloguing of the RFs (as factors of medium or high relevance) was inconclusive regarding their efficacy because a greater presence of one or the other factor was unrelated to the interventions’ effectiveness. Moreover, the factors were heterogeneously distributed in most of the programs. These findings answer the second research question of this study: Is the number of suicide RFs addressed in the programs related to the interventions’ favourable outcomes?

However, although RFs have been found to be a fundamental source for prevention, their predictive capacity is limited, and this traditional approach can be complemented by universal prevention approaches based on dynamic patterns [18]. As well as that, it is important to take into account the idea presented by “ideation-to-action” models of understanding suicide [98], which have convincingly demonstrated that suicide risk is affected not only by the desire for suicide but a capability for it as well.

This study also has some limitations: (1) the authors did not have open access to all the suicide prevention programs, and the cataloguing was done through public information; (2) only 40% of the authors of the programs agreed to provide us with additional information about their programs besides that published; (3) only programs implemented in formal education settings and targeting a specific age range were analyzed; (4) the literature analysis on systematic reviews and meta-analyses of suicide RFs was not an umbrella review (nor was it the objective of this manuscript); (5) The results of this study must be interpreted with caution since the ratio of programs analyzed has been limited by the inclusion and exclusion criteria established for the systematic review; (6) due to the study’s exclusion criteria, more current program designs that are in the implementation phase or are pending evidence of their effectiveness may have been left out.

## Practical Implications for New Prevention Programs or for Updating the Existing Ones

After all these issues, we recommend updating or creating new suicide prevention programs, preferably multi-modal ones, that address the suicide RFs prevalent in the literature (factors included in Table 1), and, especially, those common in the most effective programs: anxiety, depression, peer support, social relationships, help-seeking, and coping strategies. In terms of future measures, it is essential to develop more holistic programs that integrate a broader range of risk factors, including cyberbullying, and to provide targeted training for educators to recognize and address these issues. Furthermore, increasing collaboration between schools, mental health professionals, and parents can ensure a more comprehensive approach to prevention and intervention [99].

Likewise, work must be carried out with the family, complementing training with other universal prevention approaches based on dynamic patterns. In addition, it is urgent for future prevention programs to incorporate a holistic view of the internet risks on the one hand and, on the other hand, the prevention of school violence and a broad perspective of respect for differences and sexual orientation. These are two current problems for children and adolescents between Generation Z and Generation Alpha [100]. In short, we must rethink and update the design of suicide prevention programs for a globalized, heterogeneous, diverse, and digitalized world in need of prosocial values and attitudes, on- and offline, to promote a healthy and friendly coexistence with everyone [3]. Additionally, it is urgent to create a “National Plan for the Prevention of Suicide”, for the design of which the conclusions of this study can be taken into account, and in particular, for work in the field of formal education (this exhortation is valid for supranational levels such as the European Union). It is also crucial for the existing preventive programs to be adapted and validated in other cultural and linguistic contexts and for new programs to be implemented with a global view of their potential use in broad geographical contexts.

## Conclusions

There are many suicidal ideation RFs that should be addressed in any suicide prevention program in the child and adolescent population: anxiety, depression, peer support and social relationships. These are common variables in the most effective interventions. Likewise, there is a relationship between a higher number of suicide RFs addressed in prevention programs and their effectiveness. Sexual orientation and bullying/cyberbullying are two RFs whose role in adolescence is crucial and which are barely or not addressed in current prevention programs. Likewise, multi-modal interventions provide the best indicators of effectiveness. In addition, the inclusion of working with the family appears to be a component that affects the effectiveness of the programs. Policymakers are urged to address the need to update the existing prevention programs to meet the needs of young people (especially for Generation Z and Alpha students) in the digital society, as reality is currently co-constructed offline and online [101]. However, the approach to the topic addressed in this paper affects just to one part of the possible solutions. In this sense, simply adding more risk factors to suicide prevention programs is unlikely to have an extraordinary impact on youth suicidal behavior. Thus, the quality of implementation, such as fidelity, acceptability, appropriateness and so forth is equally important and deserves discussion as well.

## Availability of Data and Materials

All the data would be provide by the authors.

## Supplementary Material

Supplementary material associated with this article can be found, in the online version, at https://doi.org/10.62641/aep.v53i6.1938.
